# Glucocorticoids dexamethasone and prednisolone suppress fibroblast growth factor 23 (FGF23)

**DOI:** 10.1007/s00109-021-02036-8

**Published:** 2021-01-30

**Authors:** Martina Feger, Franz Ewendt, Jörg Strotmann, Holger Schäffler, Daniela Kempe-Teufel, Philipp Glosse, Gabriele I. Stangl, Michael Föller

**Affiliations:** 1 Department of Physiology, University of Hohenheim, Garbenstraße 30, 70599 Stuttgart, Germany; 2 Institute of Agricultural and Nutritional Sciences, Martin Luther University Halle-Wittenberg, 06120 Halle (Saale), Germany; 3 Department of Gastroenterology, University of Rostock, 18057 Rostock, Germany; 4 Department of Endocrinology, University of Tübingen, 72076 Tübingen, Germany

**Keywords:** Phosphate, 1,25(OH)_2_D_3_, αKlotho, Inflammation

## Abstract

**Abstract:**

Fibroblast growth factor 23 (FGF23) is a hormone mainly secreted by bone cells. Its most prominent effects are the regulation of renal phosphate reabsorption and calcitriol (active vitamin D, 1,25(OH)_2_D_3_) formation, effects dependent on its co-receptor αKlotho. Besides these actions, further paracrine and endocrine effects exist. The production of FGF23 is regulated by 1,25(OH)_2_D_3_, parathyroid hormone, dietary phosphate intake, iron status, as well as inflammation. Glucocorticoids are hormones with anti-inflammatory properties and are, therefore, widely used for acute and chronic inflammatory diseases, autoimmune disorders, and malignancies. The present study explored whether glucocorticoids influence the production of FGF23 in vitro as well as in mice*. Fgf23* transcription was analyzed by semi-quantitative real-time PCR. Serum concentrations of FGF23 and 1,25(OH)_2_D_3_ were measured by ELISA. Urinary phosphate and Ca^2+^ excretion were determined in metabolic cages. As a result, in UMR106 rat osteoblast-like cells and in MC3T3-E1 cells, both, dexamethasone and prednisolone, downregulated *Fgf23* transcription and FGF23 protein synthesis. Dexamethasone increased *Dmp1* and *Phex* (encoding FGF23-regulating genes) as well as *Nfkbia* (encoding NFκB inhibitor IκBα) transcription in UMR106 cells. In mice, a single injection of dexamethasone or prednisolone was followed by a significant decrease of serum C-terminal and intact FGF23 concentration and bone *Fgf23* mRNA expression within 12 h. These effects were paralleled by increased renal phosphate excretion and enhanced 1,25(OH)_2_D_3_ formation. We conclude that a single glucocorticoid treatment strongly downregulates the FGF23 plasma concentration.

**Key messages:**

- Glucocorticoids dexamethasone and prednisolone suppress the formation of bone-derived hormone fibroblast growth factor 23 (FGF23) in vitro.
- The effect is accompanied by an upregulation of Dmp1, Phex, and IκBα, negative regulators of FGF23, in UMR106 osteoblast-like cells.
- Glucocorticoid receptor antagonist RU-486 attenuates the effect of dexamethasone on FGF23, Dmp1, and Phex.
- In mice, a single glucocorticoid dose suppresses FGF23 and enhances 1,25(OH)_2_D_3_ (active vitamin D).

**Supplementary Information:**

The online version contains supplementary material available at 10.1007/s00109-021-02036-8.

## Introduction

The phosphaturic hormone fibroblast growth factor 23 (FGF23) is mainly synthesized in bone cells (osteoblasts/osteocytes) and affects renal phosphate handling and the production of calcitriol (1,25(OH)_2_D_3_), the active form of vitamin D [1, 2]. These effects are mediated by the concerted action of the receptor for FGF23 (FGFR) and transmembrane protein αKlotho which serves as a co-receptor [2]. Binding of FGF23 to this receptor results in the internalization of secondary-active phosphate transporter NaP_i_IIa residing in the apical cell membrane of the proximal tubule and in downregulation of *CYP27B1*, the key enzyme for the generation of 1,25(OH)_2_D_3_ [1]. Hence, FGF23 lowers the plasma concentration of both, phosphate and 1,25(OH)_2_D_3_, thereby depending on αKlotho [1], an anti-aging protein [3]. Another target organ of FGF23 is the parathyroid gland where FGF23 regulates the production of parathyroid hormone, PTH, in a αKlotho-dependent manner [4, 5]. Apart from this, FGF23 also exerts paracrine and further endocrine effects without the contribution of αKlotho: It induces hypertrophy of the left ventricle [6], regulates neutrophil recruitment [7], or stimulates the secretion of pro-inflammatory cytokines from the liver [8]. The induction of left ventricular hypertrophy may, at least in part, be due to indirect effects of FGF23, too, e.g., elevation of blood pressure [9–11]. Similarly, the FGF23 effect on inflammation in hepatocytes may also involve indirect actions, e.g., the induction of interleukin-6 production in Kupffer cells that affect hepatocytes [12].

The dramatic phenotype of FGF23- or αKlotho-deficient mice is mostly characterized by sequelae of massive hyperphosphatemia which are far-reaching and affect most organs and tissues: The animals suffer from rapid aging with a markedly shortened life span and exhibit a wide range of aging associated diseases [13, 14]. A low phosphate or low vitamin D diet prevents such consequences [15]. Similar to FGF23- or αKlotho-deficient mice, patients with chronic kidney disease (CKD), which are typically characterized by high FGF23 levels and low αKlotho expression, suffer from the sequelae of hyperphosphatemia which drastically increase their cardiovascular mortality [16]. The FGF23 plasma concentration rises early in CKD and correlates well with disease activity, progression and outcome making FGF23 a valuable disease biomarker in CKD [17, 18]. In addition, high FGF23 plasma levels are found in cardiovascular, metabolic, and inflammatory disorders, suggesting that FGF23 may be a general disease biomarker [19].

The regulation of FGF23 production is only partly understood: Important regulators include 1,25(OH)_2_D_3_ [20] and PTH [21]. Both strongly affect the plasma phosphate concentration as does FGF23. Therefore, and since FGF23 also regulates 1,25(OH)_2_D_3_ and PTH, all three hormones are part of a complex feedback loop regulating plasma phosphate. Further regulators are alimentary phosphorus intake [22], inflammation [23], and inflammatory cytokines including tumor necrosis factor-α [24], interleukin-6 [25], the iron status [26], or erythropoietin [27]. Mechanistically, the transcription of the *FGF23* gene is dependent on transcription factor Nurr1 [21], NFκB upregulating Ca^2+^ release-activated Ca^2+^ channel (CRAC) Orai1 [28, 29], as well as insulin-dependent phosphoinositide 3-kinase (PI3K)/protein kinase B (PKB)/Akt/forkhead box O1 (FOXO1) signaling [30]. Orai1-mediated store-operated Ca^2+^ entry (SOCE) is a fundamental driver of *FGF23* gene expression [28, 31]. The aforementioned signaling has, thus far, only been verified for FGF23 production in bone, not for other tissues.

Cortisol is the main glucocorticoid in humans produced by the adrenal gland [32]. Glucocorticoids are stress hormones with a long list of metabolic (particularly affecting glucose and lipid metabolism) and immunological effects [32]. Synthetic glucocorticoids such as prednisolone or dexamethasone are widely used in the therapy of inflammatory diseases owing to their strong and multiple anti-inflammatory properties [33]: Glucocorticoids inhibit the production of pro-inflammatory prostaglandins through annexins [34], induce apoptosis of T cells [35], or suppress the transcriptional activity of pro-inflammatory transcription factor complex NFκB [36]. As steroid hormones, glucocorticoids bind to an intracellular receptor, the glucocorticoid receptor (GR), which translocates to the nucleus, binds DNA, and induces or represses the transcription of target genes [37].

Glucocorticoid therapies are initiated for a plethora of frequent inflammatory and autoimmune diseases including glomerulonephritis, rheumatoid arthritis, asthma, or inflammatory bowel disease to name a few. They are limited due to a variety of adverse effects. These effects are dependent on the dose and duration of therapy, but are often observed and include steroid diabetes, hypertension, thin skin, muscle atrophy, obesity, and loss of bone mass due to direct and indirect effects [38]: Glucocorticoids affect osteoblast and osteoclast activity [39] but also impact on phosphate metabolism by regulating renal phosphate transporter NaP_i_IIa [40].

Recently, a study suggested an inhibitory effect of glucocorticoids on FGF23 [41], whereas another study suggested a stimulatory effect [42]. Both studies focused on long-term effects of glucocorticoids which may include secondary cellular responses owing to the broad spectrum of glucocorticoid effects. We therefore sought to study the cellular effects of an acute glucocorticoid treatment, both in vitro and in vivo. To this end, we analyzed the production of FGF23 in UMR106 osteoblast-like cells and in MC3T3-E1 cells as well as in mice treated with glucocorticoids.

## Materials and methods

### Cell culture

Experiments were performed in UMR106 rat osteoblastic osteosarcoma cells (CRL-1661; ATCC, Manassas, VA, USA). UMR106 cells were cultured in Dulbecco’s Modified Eagle Medium (DMEM) high glucose (Gibco, Life Technologies, Darmstadt, Germany) supplemented with 10% fetal bovine serum (FBS) (Gibco, Life Technologies), 100 U/ml penicillin, and 100 μg/ml streptomycin (Gibco, Life Technologies). Twenty-four hours after seeding, the cells were treated with the indicated concentrations of dexamethasone, prednisolone (both from Sigma-Aldrich, Schnelldorf, Germany; stock dissolved in dimethyl sulfoxide (DMSO)), or glucocorticoid receptor antagonist RU-486 (Tocris Bioscience, Bristol, UK; stock dissolved in ethanol) for 24 or 48 h, as indicated. Control cells were incubated with the appropriate volume of DMSO or ethanol (both from Sigma-Aldrich), respectively. Untreated UMR106 cells do not express appreciable amounts of *Fgf23* mRNA as reflected by the cycle threshold (Ct) of *Fgf23* in semi-quantitative RT-PCR analysis. Calcitriol potently upregulates *Fgf23* expression in UMR106 cells. In line with this, the arithmetic means ± SEM (*n* = 3) of the Ct values of UMR106 cells were 34.0 ± 0.75 (untreated) and 28.0 ± 0.06 (treated with 10 nM calcitriol for 24 h). Therefore, all cell culture treatments were carried out in the presence of 10 nM calcitriol (Tocris Bioscience) [43].

MC3T3-E1 Subclone 4 mouse pre-osteoblast cells (CRL-2593; ATCC) were cultured in alpha-Minimum Essential Medium (α-MEM) containing nucleosides and 2 mM L-glutamine (Gibco, Life Technologies) and supplemented with 10% FBS, 100 U/ml penicillin, and 100 μg/ml streptomycin. The cells were used from passages 20 to 23. The cells were seeded on rat tail type I collagen-coated 12-well plates (80,000 cells per well) for 24 h. Differentiation was induced by culturing the cells in osteogenic medium containing 50 μg/ml ascorbic acid (Sigma-Aldrich), and 4 mM β-glycerophosphate (AppliChem, Darmstadt, Germany). Cells were differentiated for 6 or 13 days and then treated for 24 h with dexamethasone (30 nM or 100 nM) or vehicle in the presence of calcitriol (10 nM) throughout the experiment or 24 h before harvesting the cells, as indicated.

### Analysis of FGF23 protein in the cell culture medium

The cell culture supernatants of UMR106 cells treated with 30 nM or 100 nM dexamethasone or vehicle in the presence of 10 nM calcitriol for 48 h were collected and stored at −70 °C. The medium was concentrated utilizing Vivaspin 6 concentrators (Sartoritus, Göttingen, Germany). C-terminal FGF23 and intact FGF23 were determined by commercial ELISA Kits (Mouse/Rat FGF-23 (C-Term), Mouse/Rat FGF-23 (Intact); both from Immutopics, San Clemente, CA, USA) according to the manufacturer’s instructions.

### Animal experiments and tissue collection

All animal experiments were conducted in accordance with the federal law for the welfare of animals and were approved by the respective state government. The mice had free access to tap water and standard rodent chow mainly based on wheat, soybeans, barley, oat hulls, and sugar beet pulp with a rough composition owing to the nature of the plant products (V1534: app. 0.7% phosphorus, 1.0% calcium; Ssniff, Soest, Germany; more details can be found at https://www.ssniff.de/) ad libitum.

Adult wild-type mice (C57BL/6; Charles River, Sulzfeld, Germany) of both sexes were injected intraperitoneally with a single dose of dexamethasone (20 mg/kg body weight; Fortecortin®, Merck, Darmstadt, Germany; formulation: 4.37 mg/ml dexamethasone dihydrogen phosphate disodium, sodium edetate, creatinine, sodium citrate, 1 mol/l sodium hydroxide and water for injections) or prednisolone (20 mg/kg body weight; PredniGalen®, GALENpharma, Kiel, Germany; formulation: 10 mg/ml prednisolone acetate, macrogol 4000, polysorbate 80, disodium phosphate dodecahydrate, sodium dihydrogen phosphate dihydrate, water for injections and 15 mg/ml benzyl alcohol). As control, 0.9% saline was chosen since an exact formulation of the drugs was not available. After 12 h, blood was taken by retro-orbital puncture under isoflurane anesthesia. The animals were sacrificed and kidneys were immediately isolated and snap-frozen in liquid nitrogen. Bones were also removed and prepared by thoroughly scrubbing the femur and tibia to remove other tissues. Next, both epiphyses were cut, and the bone marrow was flushed out with 0.9% saline.

### Serum biochemistry

Photometric methods were used for the analysis of the serum phosphate (Analyticon Biotechnologies, Lichtenfels, Germany) and Ca^2+^ (Fuji DRI-CHEM NX500i, Fujifilm, Düsseldorf, Germany) concentration. For the serum concentration of 1,25(OH)_2_D_3_ (IDS, Boldon, UK), C-terminal FGF23, intact FGF23, and intact PTH (all three from Immutopics) ELISA kits were employed.

### Renal parameters

To assess renal phosphate and Ca^2+^ excretion, the mice were placed individually in siliconized metabolic cages (Tecniplast, Hohenpeissenberg, Germany). Standard chow pellets tend to crumble when eaten by mice. Hence, they cannot be used in metabolic cages since they would contaminate the collected urine. This is why we used a control diet with pellets with different consistency and precisely defined ingredients (C1000: 0.8% phosphorus, 0.9% calcium; Altromin, Lage, Germany; more detailed information can be found at https://altromin.de/). Following a 3-day habituation period, mice were treated with 20 mg/kg body weight dexamethasone or vehicle. After 12 h, a small amount of blood was withdrawn. Additionally, 24-h urine was collected under water-saturated oil starting from the time of injection. The urinary phosphate and Ca^2+^ concentrations were measured by means of commercial assays (both from Analyticon Biotechnologies, Lichtenfels, Germany). Prior to the determination of Ca^2+^, 24-h urine was acidified with hydrochloric acid in order to dissolve calcium salts. A creatinine detection kit (Jaffé method, Labor + Technik, Berlin, Germany) was employed for the measurement of the urinary creatinine concentration. Serum creatinine was determined by an enzymatic method (Chrystal Chem, Zaandam, Netherlands).

### Semi-quantitative real-time PCR

Total RNA was extracted from UMR106 cells, MC3T3-E1 cells, or mouse kidneys using peqGold Trifast™ reagent (Peqlab, Erlangen, Germany). Total RNA (1.2 μg) was reverse-transcribed with the GoScript™ Reverse Transcription System (Promega, Mannheim, Germany) and random primers (Promega). One μg of total RNA was used for first-strand cDNA from MC3T3-E1 cells with persistent calcitriol stimulation.

Total bone RNA was initially extracted with peqGold Trifast™ reagent followed by a DNase treatment and a purification step using the RNase-free DNase Set and the RNeasy Mini Kit (both from QIAGEN, Hilden, Germany). First-strand cDNA was synthesized from 300 ng of total bone RNA using the GoScript™ Reverse Transcription System.

For the analysis of relative transcript levels, semi-quantitative real-time PCR (qRT-PCR) using GoTaq® qPCR Master Mix (Promega) was carried out. The reaction mix for the amplification of 2 μl cDNA templates (1.5 μl cDNA in the case of bone *Fgf23* expression analysis) contained 10 μl 2x GoTaq® qPCR Master Mix (Promega), primers, and sterile water up to 20 μl. The qRT-PCR program was 95 °C for 2 min, 40 cycles of denaturation at 95 °C for 10 s, annealing at primer-specific temperature for 30 s, and extension at 72 °C for 25–30 s.

The following primers (5′ → 3′ orientation) and annealing temperatures were used:

*rat Dmp1* (62 °C):

CGCCCATGGCAAATAGTGAC;

CGTGCTGTCTTCACTGGACT;

*rat Fgf23* (57 °C):

TAGAGCCTATTCAGACACTTC;

CATCAGGGCACTGTAGATAG;

*rat Nfkbia* (58 °C):

AGACTCGTTCCTGCACTTGG;

TCTCGGAGCTCAGGATCACA;

*rat Phex* (58 °C):

ATGGCTGGATAAGCAATAAC;

GCTTTTTCAATCGCTTTCTC;

*rat Tbp* (57 °C):

ACTCCTGCCACACCAGCC;

GGTCAAGTTTACAGCCAAGATTCA;

*mouse Cyp24a1* (58 °C):

CCCAAAGGAACAGTCTTAAC;

GGTCTAAACTTGTCAGCATC;

*mouse Cyp27b1* (58 °C):

AGTGTTGAGATTGTACCCTG;

CGTATCTTGGGGAATTACATAG;

*mouse Dmp1* (58 °C):

GAACAGTGAGTCATCAGAAG;

AAAGGTATCATCTCCACTGTC;

*mouse Fgf23* (58 °C):

TCGAAGGTTCCTTTGTATGGA;

AGTGATGCTTCTGCGACAAGT;

*mouse Gapdh* (58 °C):

GGTGAAGGTCGGTGTGAACG;

CTCGCTCCTGGAAGATGGTG;

*mouse K1* (56 °C):

CCTTAAAAGCAATCAGACTGG;

GAAAGCCATTGTCCTCTATC;

*mouse Nfkbia* (57 °C):

GGAGACTCGTTCCTGCACTTGG;

CTCAGCAATTCCTGGCTGGT;

*mouse Phex* (57 °C):

TCATTGATACCAGACTCTACC;

CAATGGTTTTCTTCCTCTCG;

*mouse Slc34a1* (58 °C):

AATGCAACCATATCTTCGTG;

GGAAAGTCTGTGTTGATGAC;

*mouse Tbp* (60 °C):

CCAGACCCCACAACTCTTCC;

CAGTTGTCCGTGGCTCTCTT.

Calculated mRNA expression levels were normalized to the expression levels of *Tbp* (rat UMR106 cells and mouse MC3T3-E1 cells) and *Gapdh* (mouse tissue). Relative quantification of gene expression was performed using the 2^-ΔΔCt^ method.

### Statistics

Data are shown as arithmetic means ± SEM, and *n* represents the number of mice per group or the number of independent cell culture experiments, respectively. Normality was tested with Shapiro-Wilk test. Two groups were compared by paired *t* test or unpaired two-tailed *t* test (Welch’s correction was applied for data with different standard deviations) or Mann-Whitney U test (for data not passing normality test). Statistical testing of more than two groups was performed by one-way ANOVA followed by Dunnett’s or Tukey’s multiple comparisons test (homoscedastic data) or Games-Howell test (heteroscedastic data) as indicated in the figure legends. Only differences with *P* < 0.05 were considered statistically significant.

## Results

**Fig. 1:**
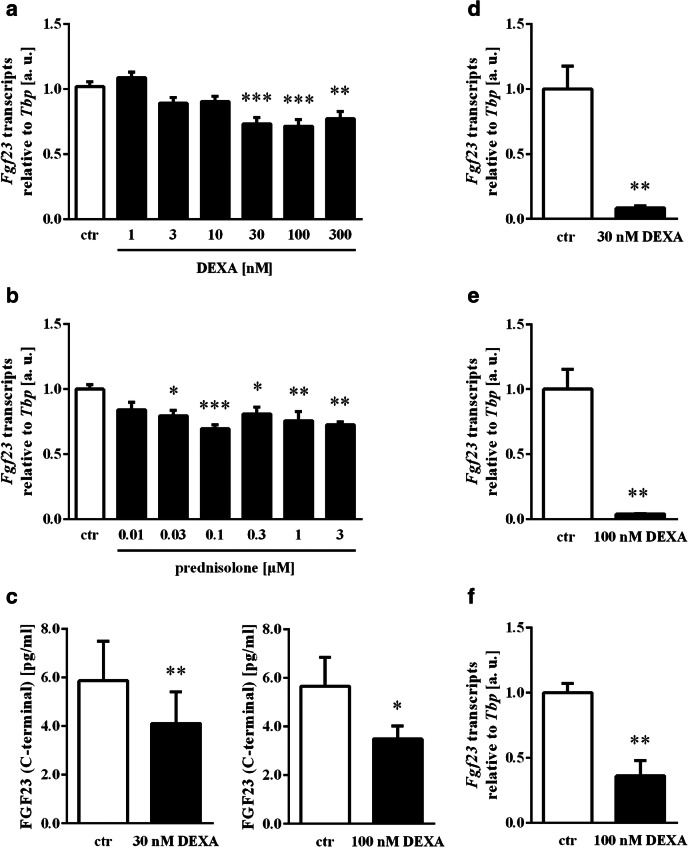
Effect of glucocorticoids on *Fgf23* expression and protein synthesis in UMR106 and MC3T3-E1 cells. Arithmetic means ± SEM (*n* = 6, arbitrary units (a. u.)) of relative *Fgf23* mRNA expression normalized to *Tbp* transcript levels in UMR106 cells incubated without (white bars) or with (black bars) dexamethasone (DEXA, **a**) or prednisolone (**b**) at the indicated concentrations in the presence of calcitriol (10 nM) for 24 h. *p < 0.05, **p < 0.01, ***p < 0.001 indicate significant differences compared to UMR106 cells treated with 10 nM calcitriol only. (**c**) Arithmetic means ± SEM of C-terminal FGF23 protein concentration in the cell culture supernatant of UMR106 cells treated with vehicle (white bars) or with 30 nM (*n* = 8) or 100 nM (n = 6) dexamethasone (DEXA, black bars) in the presence of calcitriol (10 nM) for 48 h. *p < 0.05, **p < 0.01, indicate significant difference compared to UMR106 cells treated with 10 nM calcitriol only. Arithmetic means ± SEM (*n* = 5, arbitrary units (a. u.)) of relative *Fgf23* mRNA expression normalized to *Tbp* transcript levels in MC3T3-E1 cells at day 7 of osteoblast differentiation incubated with vehicle (white bars) or 30 nM (**d**) or 100 nM (**e**) dexamethasone (DEXA, black bars) in the presence of calcitriol (10 nM) for 24 h. **p < 0.01 indicates significant difference compared to control cells. (**f**) Arithmetic means ± SEM (*n* = 4, arbitrary units (a. u.)) of relative *Fgf23* mRNA expression normalized to *Tbp* transcript levels in MC3T3-E1 cells at day 14 in osteogenic differentiation medium containing calcitriol (10 nM) and incubated with 100 nM dexamethasone (DEXA, black bar) or vehicle (white bar) for 24 h. **p < 0.01 indicates significant difference compared to vehicle-treated MC3T3-E1 cells. (**a** and **b**: one-way ANOVA followed by Dunnett’s multiple comparisons test; **c–f**: paired *t* test)

We used UMR106 osteoblast-like cells to study whether dexamethasone, a synthetic glucocorticoid with high potency that is widely used in clinical practice, influences FGF23 production. The cells were incubated with 1–300 nM dexamethasone for 24 h, and *Fgf23* transcripts were determined by qRT-PCR. The dose dependence of the dexamethasone effect on *Fgf23* gene expression is depicted in Fig. 1a. Similarly, the expression of *Fgf23* mRNA was reduced by the synthetic glucocorticoid prednisolone that is also widely used in the treatment of autoimmune diseases (Fig. 1b). In order to test whether dexamethasone also affects FGF23 secretion, we analyzed the FGF23 protein concentration into the cell culture supernatant. As a result, the concentration of C-terminal FGF23 in the cell culture medium of cells incubated with 30 nM (Fig. 1c, left panel) or 100 nM (Fig. 1c, right panel) dexamethasone for 48 h was significantly lower than in the medium of vehicle-treated cells. We also tried to determine intact FGF23, however, for *n* = 7 out of *n* = 14 experiments, the concentration of intact FGF23 was below the detection limit. For the remaining *n* = 7 measurements, the concentration of intact FGF23 was 3.49 ± 0.51 pg/ml in the supernatant of UMR106 cells incubated for 48 h without and 2.52 ± 0.21 pg/ml in the supernatant of cells incubated with 30 nM dexamethasone (*p* = 0.12).

MC3T3-E1 cells were used in further experiments to verify the glucocorticoid effect on FGF23. Also in these cells dexamethasone potently lowered *Fgf23* gene expression (Figs. 1d–f). The effect could be observed in cells differentiated for 6 days (Figs. 1d, e) or 13 days (Fig. 1f) and then treated with 30 nM (Fig. 1d) or 100 nM (Figs. 1e, f) dexamethasone for 24 h.

**Fig. 2:**
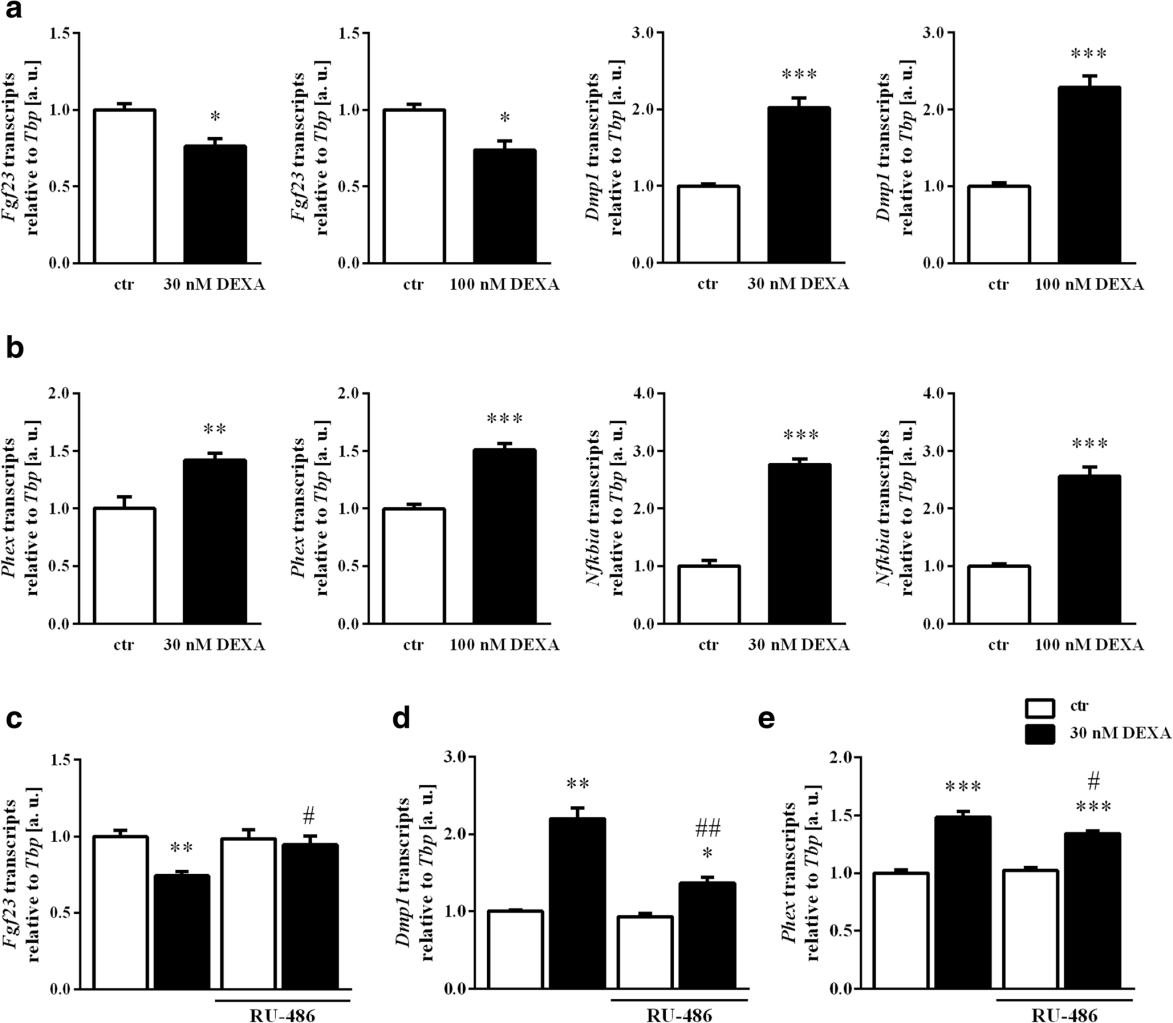
Impact of dexamethasone and glucocorticoid antagonist RU-486 on regulators of *Fgf23* expression. Arithmetic means ± SEM (arbitrary units (a. u.)) of relative mRNA transcript levels of *Fgf23*, *Dmp1* (**a**), *Phex*, and *Nfkbia* (**b**), all normalized to *Tbp,* in UMR106 cells treated with 30 nM (*n* = 9) or 100 nM dexamethasone (*n* = 7) (DEXA, black bars) or vehicle only (white bars) in the presence of 10 nM calcitriol for 24 h. *p < 0.05, **p < 0.01, ***p < 0.001 indicate statistically significant difference from vehicle-treated cells. Arithmetic means ± SEM (n = 6, arbitrary units (a. u.)) of relative mRNA transcript levels of *Fgf23* (**c**), *Dmp1* (**d**), and *Phex* (**e**), all normalized to *Tbp* in calcitriol-stimulated (10 nM for 24 h) UMR106 cells treated without (white bars) or with 30 nM dexamethasone (DEXA, black bars) in the absence or presence of the glucocorticoid receptor antagonist RU-486 (30 nM) for 24 h. *p < 0.05, **p < 0.01, ***p < 0.001 indicate statistically significant difference from vehicle-treated cells (first bar). #*p* < 0.05, ##*p* < 0.01 indicate statistically significant difference from the absence of RU-486 (2^nd^ bar vs. 4^th^ bar). (**a** and **b**: paired *t* test; **c–e**: one-way ANOVA followed by Tukey’s multiple comparisons test (**c** and **e**) or Games-Howell test (**d**))

A new series of experiments addressed the impact of dexamethasone on regulators of *Fgf23* expression. Again, 30 nM or 100 nM dexamethasone suppressed *Fgf23* gene expression in UMR106 cells (Fig. 2a). Importantly, *Dmp1* (Fig. 2a, right panels) and *Phex* (Fig. 2b, left panels) mRNA transcript levels were significantly higher in dexamethasone-treated (30 nM or 100 nM) UMR106 cells than in control cells. Glucocorticoids have strong anti-inflammatory properties owing to their inhibitory effect on NFκB. Hence, we studied the expression of IκBα (encoded by *Nfkbia*), a cytoplasmic protein that inhibits nuclear translocation of NFκB. As shown in Fig. 2b, right panels, *Nfkbia* expression was significantly enhanced following treatment with 30 or 100 nM dexamethasone for 24 h*.*

We also investigated the contribution of the glucocorticoid receptor to the dexamethasone effect on FGF23 by performing experiments with glucocorticoid receptor antagonist RU-486. As a result, 30 nM RU-486 significantly attenuated the dexamethasone (30 nM) effect on *Fgf23* transcripts (Fig. 2c), *Dmp1* transcripts (Fig. 2d) and *Phex* transcripts (Fig. 2e). Thus, the dexamethasone effect is—at least in part—mediated by the glucocorticoid receptor.

Since the cells had to be treated with 1,25(OH)_2_D_3_ to upregulate *Fgf23* gene expression, the dexamethasone effect could, at least in theory, have been due to an interaction of dexamethasone with 1,25(OH)_2_D_3_ signaling. Therefore, we tested whether the dexamethasone effect on upstream regulators of *Fgf23* gene expression was different between cells treated with 1,25(OH)_2_D_3_ or left untreated. As demonstrated in suppl. Fig. 1, the effect of 30 nM dexamethasone on *Dmp1* expression (suppl. Fig. 1a), *Phex* expression (suppl. Fig. 1b), or *Nfkbia* expression (suppl. Fig. 1c) was comparable between cells treated with 1,25(OH)_2_D_3_ or left untreated.

**Fig. 3:**
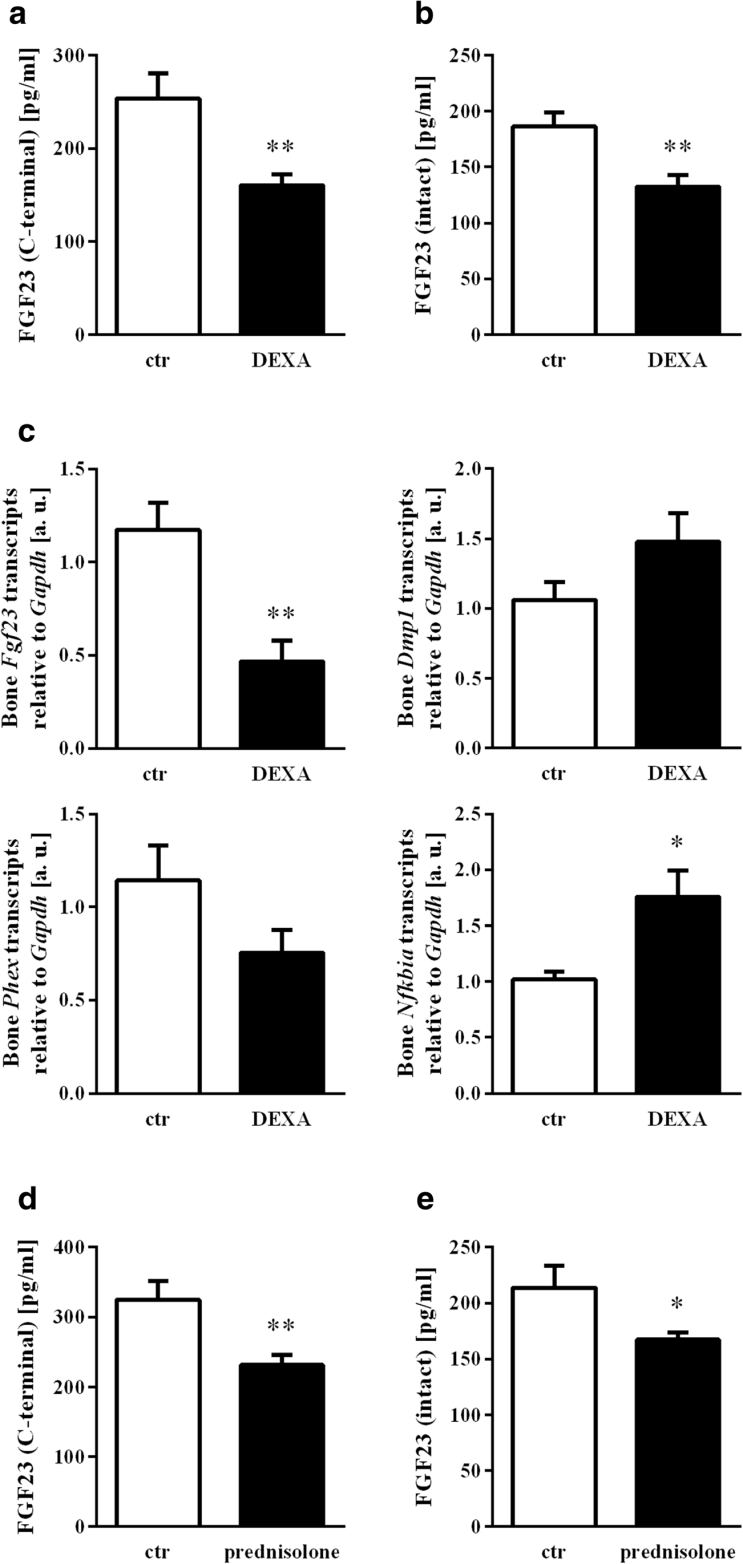
The glucocorticoid effect on serum FGF23 and bone *Fgf23* expression. Arithmetic means ± SEM of the serum concentration of C-terminal FGF23 (**a**, *n* = 11) and intact FGF23 (**b**, *n* = 13) as well as relative osseous mRNA expression (**c**) of *Fgf23* (*n* = 7, arbitrary units (a. u.), *Dmp1* (n = 9), *Phex* (n = 9), and *Nfkbia* (n = 9) normalized to housekeeping gene *Gapdh* in control mice (ctr, white bars) and dexamethasone-treated mice (20 mg/kg body weight, DEXA, black bars) 12 h after injection. Arithmetic means ± SEM of the serum concentration of C-terminal (**d**, *n* = 10) and intact FGF23 (**e**, *n* = 10) in mice 12 h after treatment with vehicle (ctr, white bars) or prednisolone (20 mg/kg body weight; black bars). *p < 0.05, **p < 0.01 indicate significant difference compared to vehicle-treated mice. (**a**, **c** (right lower panel) and **e**: unpaired *t* test with Welch’s correction; **b** and **c** (right upper panel): unpaired *t* test; **c** (left upper panel and left lower panel) and **d**: Mann-Whitney U test)

Our in vitro results suggest an inhibitory effect of glucocorticoids on FGF23 synthesis. We therefore sought to investigate whether the regulation of FGF23 by glucocorticoids has in vivo relevance given their frequent use for medical purposes. Mice were sham-treated or treated with a single dose of dexamethasone. Twelve hours later, blood was collected, and the serum concentration of C-terminal and intact FGF23 was determined. As demonstrated in Fig. 3, dexamethasone is given only once significantly and markedly reduced the serum level of both, C-terminal (Fig. 3a) and intact (Fig. 3b) FGF23. To check whether the lower FGF23 serum concentration is paralleled by reduced gene expression, we determined *Fgf23* transcript levels in the bone of dexamethasone-treated animals, the main site of FGF23 production. As a result, dexamethasone downregulated *Fgf23* gene expression in the bone, pointing to a highly relevant inhibitory effect of glucocorticoids on the production of this hormone even after a single dose (Fig. 3c, first panel). The effect was paralleled by upregulation of *Nfkbia* expression (Fig. 3c, last panel), whereas *Dmp1* and *Phex* expressions were not significantly modified (Figs. 3c, 2^nd^ and 3^rd^ panel). Moreover, a single injection of prednisolone similarly lowered the C-terminal (Fig. 3d) and intact FGF23 serum level (Fig. 3e).

**Fig. 4:**
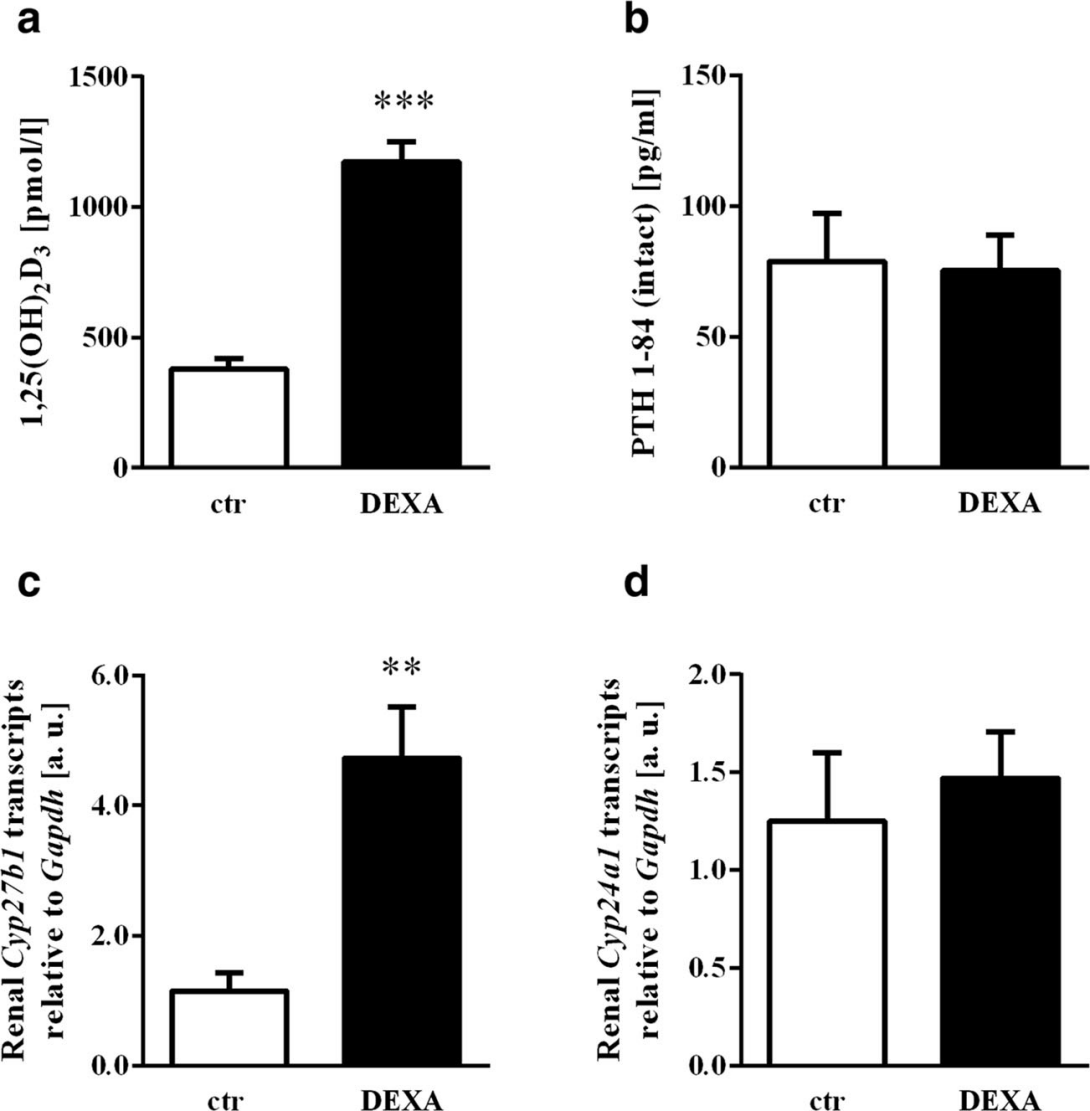
Impact of dexamethasone on 1,25(OH)_2_D_3_ formation, metabolism and PTH serum level. Arithmetic means ± SEM (*n* = 6) of the 1,25(OH)_2_D_3_ (**a**) and intact PTH (**b**) serum concentration in control mice (ctr, white bars) and mice treated with 20 mg/kg body weight dexamethasone (DEXA, black bars) 12 h after the injection. Arithmetic means ± SEM (*n* = 6, arbitrary units (a. u.)) of relative mRNA transcript levels of *Cyp27b1* (**c**) and *Cyp24a1* (**d**) normalized to *Gapdh* in kidneys from mice 12 h after the injection of vehicle only (ctr, white bars) or 20 mg/kg body weight dexamethasone (DEXA, black bars). **p < 0.01, ***p < 0.001 indicate statistically significant differences from control-treated mice. (**a**, **b** and **d**: unpaired *t* test; **c**: unpaired *t* test with Welch’s correction)

One of the main actions of FGF23 is the suppression of the formation of 1,25(OH)_2_D_3_ by downregulation of *CYP27B1*, the key renal enzyme for the hydroxylation of 25(OH)D_3_. Dexamethasone-dependent inhibition of FGF23 formation is therefore likely to also affect the serum concentration of 1,25(OH)_2_D_3_. As expected, the single dose of dexamethasone resulted in a surge of the serum concentration of 1,25(OH)_2_D_3_ (Fig. 4a). PTH was not affected by dexamethasone treatment (Fig. 4b). Enhanced 1,25(OH)_2_D_3_ formation upon short-term glucocorticoid administration was paralleled by increased renal expression of *Cyp27b1* (Fig. 4c). The expression of *Cyp24a1*, the renal enzyme catalyzing the inactivation of 1,25(OH)_2_D_3_ was, however, not significantly affected by dexamethasone (Fig. 4d).

**Fig. 5:**
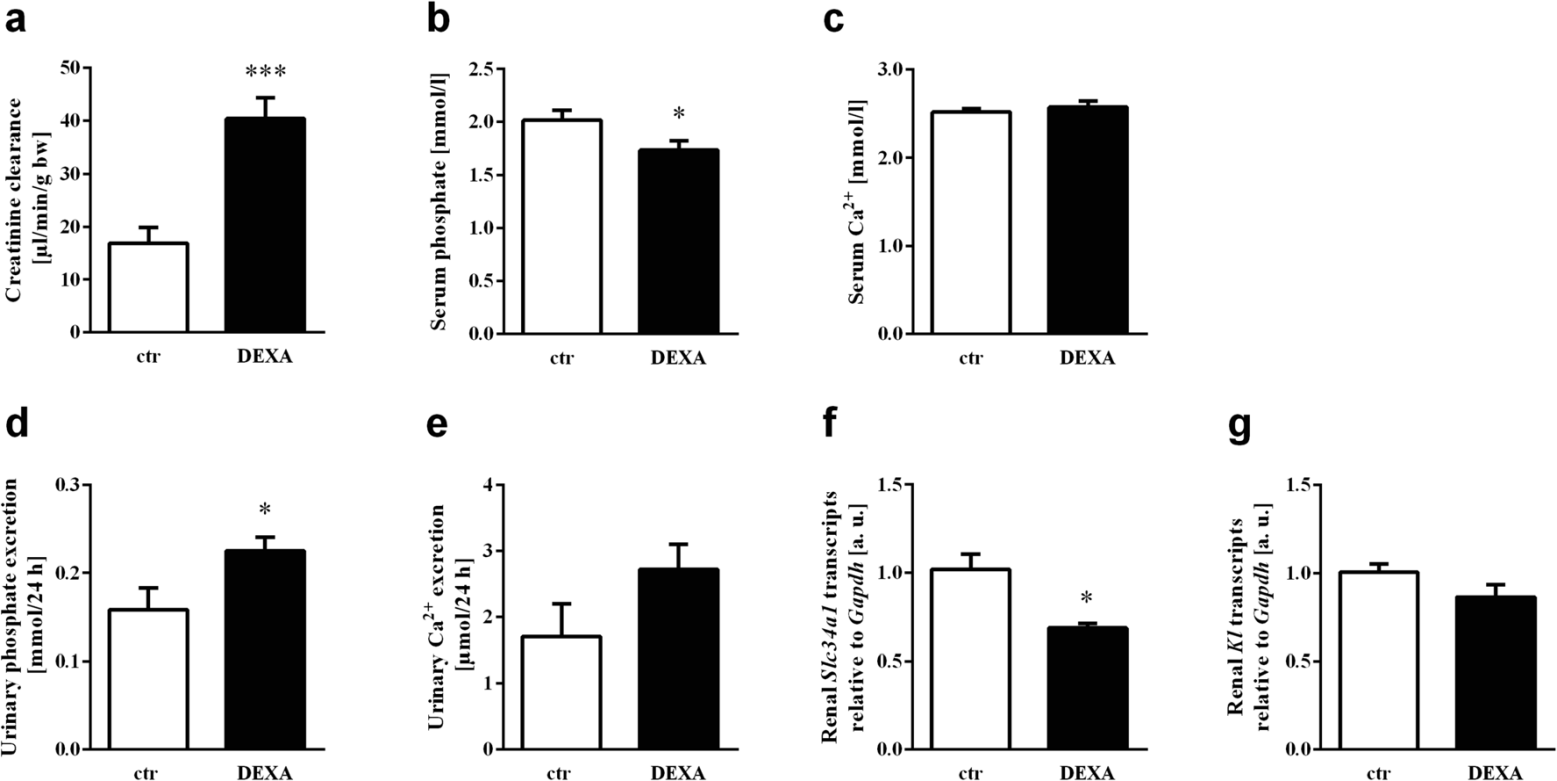
Effect of dexamethasone on phosphate and Ca^2+^ metabolism. Arithmetic means ± SEM of creatinine clearance (**a**, *n* = 6), the serum concentration of phosphate (**b**, *n* = 17; 12 h after injection) and Ca^2+^ (**c**, *n* = 12; 12 h after injection), as well as phosphate (**d**, *n* = 6) and Ca^2+^ (**e**, *n* = 6) excretion in mice in 24 h urine after injection of vehicle (ctr, white bars) or 20 mg/kg body weight dexamethasone (DEXA, black bars). Arithmetic means ± SEM (*n* = 6, arbitrary units (a. u.)) of relative mRNA transcript levels of *Slc34a1* (**f**) and *Kl* (**g**) normalized to *Gapdh* in kidneys from mice 12 h after the injection of vehicle (ctr, white bars) or 20 mg/kg body weight dexamethasone (DEXA, black bars). *p < 0.05, ***p < 0.001 indicate statistically significant differences from control mice. (**a, b, d**, **e** and **g**: unpaired *t* test; **c**: Mann-Whitney U test; **f**: unpaired *t* test with Welch’s correction)

FGF23 directly induces renal phosphate excretion by targeting NaP_i_IIa (encoded by *Slc34a1*), the main renal Na^+^-dependent phosphate transporter. To study the impact of a single dose of dexamethasone on renal phosphate handling, metabolic cage studies were conducted. Dexamethasone significantly increased creatinine clearance (Fig. 5a), a finding in line with the literature [44]. As demonstrated in Fig. 5b and d, dexamethasone treatment resulted in an increase in renal phosphate excretion, which was paralleled by a lower serum phosphate level, within 12 h. This finding is also in line with an earlier report demonstrating a direct effect of dexamethasone on NaP_i_IIa [40]. The serum Ca^2+^ concentration (Fig. 5c) was not affected by the glucocorticoid within 12 h, whereas dexamethasone treatment tended to increase urinary Ca^2+^ excretion; however, this effect did not reach statistical significance (Fig. 5e). In line with phosphaturia upon dexamethasone treatment, the renal expression of *Slc34a1* was reduced in animals receiving the glucocorticoid (Fig. 5f), as revealed by qRT-PCR. FGF23-mediated regulation of urinary phosphate excretion is dependent on its co-receptor αKlotho. As shown in Fig. 5g, treatment with dexamethasone did not significantly affect *Kl* mRNA expression in the kidney.

## Discussion

Our study discloses glucocorticoids as powerful suppressors of the production of FGF23. We demonstrated in vitro and in vivo that the widely used glucocorticoids dexamethasone and prednisolone downregulate *Fgf23* gene expression and lower the serum concentration of FGF23.

Dexamethasone is a synthetic glucocorticoid with high potency that is widely used in the treatment of inflammatory conditions, but also in cerebral edema and to prevent chemotherapy-induced nausea and vomiting [38]. In contrast to some other glucocorticoids, it has no considerable mineralocorticoid activity [45]. Prednisolone is widely used in autoimmune diseases including rheumatoid arthritis, polymyalgia rheumatica, or inflammatory bowel disease. Mineralocorticoid receptor agonists including aldosterone induce the synthesis of FGF23, as seen in primary and secondary hyperaldosteronism [46]. In contrast, our study reveals the GR as a negative regulator of FGF23.

The regulation of renal Na^+^-dependent phosphate transport by dexamethasone has been described before [40]. In their study, an inhibitory effect on the membrane expression of NaP_i_ paralleled by phosphaturia was found after 4 days of dexamethasone treatment [40]. We also observed phosphaturia in response to acute dexamethasone treatment. It is therefore possible that, apart from direct suppression of FGF23 as suggested by our cell culture experiments, dexamethasone-induced phosphaturia also contributes to the suppression of FGF23 production.

We observed a strong and significant reduction of the FGF23 serum concentration as early as 12 h after a single dose of dexamethasone or prednisolone. Notably, both, C-terminal and intact FGF23 were reduced, suggesting that glucocorticoids indeed suppress *Fgf23* gene expression rather than merely affecting post-translational processing. The latter would have been expected if C-terminal FGF23 had only been affected by the glucocorticoid. Short-term treatments even with very high doses of glucocorticoids are frequent in daily clinical practice. Therefore, our results may be of high relevance and glucocorticoid-dependent suppression of FGF23 a frequent phenomenon.

Due to the inhibitory effect of FGF23 on the formation of 1,25(OH)_2_D_3_, dexamethasone-dependent suppression of FGF23 could be expected to elevate the 1,25(OH)_2_D_3_ serum concentration. In line with this, we saw a surge in the 1,25(OH)_2_D_3_ serum level 12 h after the single dose of dexamethasone. In contrast, it is well-established textbook knowledge that particularly a long-term glucocorticoid therapy clinically indicated in various chronic autoimmune diseases requires the patient to take additional vitamin D to prevent bone loss [47], and steroid therapy is indeed associated with low 25(OH)D_3_ levels [48]. A glucocorticoid-induced increase in active vitamin D may therefore come as a surprise. It is, however, the logical consequence of the suppression of FGF23 formation by glucocorticoids. In addition, it must be kept in mind that our study only reflects the acute effects of a single dose of glucocorticoids within 12 h. It is well conceivable that the immediate increase in the serum 1,25(OH)_2_D_3_ concentration due to a glucocorticoid-dependent downregulation of FGF23 is overridden by further direct and indirect effects of glucocorticoids on the bone and vitamin D metabolism in the long run resulting in the well-documented bone loss and vitamin D deficiency.

According to our results, neither Ca^2+^ metabolism nor PTH secretion was acutely affected by glucocorticoid administration. In contrast, a long lasting steroid therapy favors renal Ca^2+^ loss and secondary hyperparathyroidism [49]. It appears to be likely that the acute effect of a glucocorticoid is therefore the downregulation of FGF23, whereas other effects on bone and Ca^2+^ and phosphate metabolism come later. Similar to the cell culture experiments, *Nfkbia* was upregulated in vivo, whereas *Dmp1* and *Phex* were not significantly affected by dexamethasone treatment. In our in vivo study, glucocorticoid effects on blood parameters were determined after 12 h whereas urine was collected for 24 h in order to cover an entire day-night period. Hence, this may be a limitation of our study since glucocorticoids may affect circadian rhythm as well as urinary Ca^2+^ and phosphate excretion may be influenced by circadian rhythm. For technical reasons, pellets with different consistency had to be used in the metabolic cages. This diet had a slightly different composition compared to standard chow. However, since all ingredients were present in adequate amounts in both diets, this difference is unlikely to significantly affect the results.

A short-term glucocorticoid treatment is particularly relevant when strong anti-inflammatory (e.g., anaphylactic shock) or anti-emetic effects (e.g., chemotherapy) are needed. According to the results of this study, such a therapy could be paralleled by a decrease of FGF23 and a surge in 1,25(OH)_2_D_3_. The exact relevance of these anticipated changes in blood parameters needs to be defined in future clinical studies. However, it is intriguing to speculate that a decrease in the FGF23 serum level may indeed be beneficial as FGF23 has been shown to go up in various cardiovascular and renal diseases and in particular in those associated with inflammation.

Our cell culture studies disclosed that dexamethasone and prednisolone suppress *Fgf23* gene expression in two bone cell lines. Moreover, this effect was paralleled by a lower C-terminal FGF23 protein concentration in the cell culture supernatant, i.e., secretion of FGF23 was also reduced by dexamethasone. Also the concentration of intact FGF23 tended to be lower in the supernatant from dexamethasone-treated cells although the values were below the detection limit for 50% of the samples. In both cell lines, *Fgf23* gene expression was induced by 1,25(OH)_2_D_3_ as described by others before [43]. The glucocorticoid effect on FGF23 could have been, at least in part, due to the crosstalk of 1,25(OH)_2_D_3_ and glucocorticoid signaling. However, the dexamethasone effect on upstream regulators of *Fgf23* gene expression *Dmp1*, *Phex*, or *Nfkbia* was not altered by 1,25(OH)_2_D_3_. Therefore, it appears to be likely that glucocorticoids are not merely effective through suppression of 1,25(OH)_2_D_3_ signaling. However, these findings do not completely rule out that dexamethasone also downregulates 1,25(OH)_2_D_3_ signaling, at least to some extent. It must be kept in mind that the in vivo experiments clearly indicate that glucocorticoids indeed suppress FGF23, a finding suggesting that also the cell culture experiments reveal a relevant effect of glucocorticoids on FGF23.

Many of the multiple cellular effects of glucocorticoids can directly or indirectly be referred to their anti-inflammatory properties that are also the reason for their therapeutic potential in various diseases. Notably, inflammation in the form of transcription factor complex NFκB is a major driver of FGF23 production [28]. In an attempt to further characterize the molecular mechanism of the dexamethasone effect on FGF23 we found that dexamethasone induces gene expression of IκBα, an inhibitor of NFκB that mediates anti-inflammatory effects of glucocorticoids [36]. Moreover, our experiments revealed that gene expression of FGF23 inhibitors dentin matrix acidic phosphoprotein 1 (Dmp1) and phosphate regulating endopeptidase homolog X-linked (Phex) [50] are also upregulated by dexamethasone [51] as has been shown by others before [52, 53]. Hence, the glucocorticoid-dependent inhibition of FGF23 is likely to be, at least in part, mediated by the glucocorticoid effects on IκBα, Dmp1, and Phex. Using glucocorticoid receptor antagonist RU-486, we could further demonstrate that the dexamethasone effects were, at least in part, mediated by the glucocorticoid receptor.

Taken together, our study demonstrates that glucocorticoids acutely downregulate the production of FGF23.

## Supplementary Information

ESM 1 (DOCX 71.5 kb)

## Data Availability

The datasets generated during and/or analyzed during the current study are available from the corresponding author on reasonable request.
