# Supplementary material for: Glucocorticoids dexamethasone and prednisolone suppress fibroblast growth factor 23 (FGF23)
Source: J Mol Med (Berl). 2021 Jan 30;99(5):699–711. doi: 10.1007/s00109-021-02036-8 (PMC8055636; doi:10.1007/s00109-021-02036-8)
Supplement: Supplementary file 1 — (DOCX 71.5 kb) [file 109_2021_2036_MOESM1_ESM.docx]

**Supplemental Figure**

**

**

**Suppl. Figure 1. Impact of 1,25(OH)_2_D_3_ on the dexamethasone effect on *Dmp1*, *Phex*, and *Nfkbia* gene expression.**

Arithmetic means ± SEM (n = 6, arbitrary units (a. u.)) of relative mRNA transcript levels of *Dmp1* (**a**), *Phex* (**b**), and *Nfkbia* (**c**), all normalized to *Tbp*, in UMR106 cells treated with 30 nM dexamethasone (DEXA, black bars) or vehicle only (white bars) in the absence (left panel) or presence of 10 nM 1,25(OH)_2_D_3_ (right panel) for 24 h. ***p* < 0.01, ****p* < 0.001 indicate statistically significant difference from control-treated cells. (**a**-**c**: paired *t* test)
